# Editorial: Phenotyping mouse embryos

**DOI:** 10.3389/fcell.2023.1284433

**Published:** 2023-09-05

**Authors:** Stefan H. Geyer, Dorota Szumska, Gabriel G. Martins, Wolfgang J. Weninger

**Affiliations:** ^1^ Division of Anatomy, Center for Anatomy and Cell Biology, Medical Imaging Cluster, Medical University of Vienna, Vienna, Austria; ^2^ Department of Physiology, Anatomy and Genetics (DPAG), and the Institute of Developmental and Regenerative Medicine (IDRM), University of Oxford, Oxford, United Kingdom; ^3^ Instituto Gulbenkian de Ciência (IGC), Oeiras, Portugal

**Keywords:** mouse embryo, phenotyping, imaging, mouse model, Morphogenesis

The mouse shares many morphological and genetic similarities with humans, making a valuable experimental model for studying human disease. Molecular tools to modify the mouse genome have been developed and refined over decades and with the advent of the CRISPR-Cas9 and similar technologies, targeted manipulations have become standard. In parallel, a wide spectrum of sophisticated morphological and functional imaging methods emerged recently, that allow precise two-dimensional (2D), three-dimensional (3D) and even four-dimensional (4D) visualisation of organs and tissues, often combined with the molecular and structural environments of cells and the intercellular space. Together, this enables examining the complex regulation of processes in ontogenesis and the mechanisms of tissue remodelling in mammals. This, in turn, is essential for understanding the principles of morphogenetic and physiological processes in humans and researching the causality of hereditary diseases.

In this Research Topic we aimed to collect Original Articles and Reviews, that introduce the reader to state-of-the-art imaging methods for studying the phenotype of normal and genetically engineered mouse embryos.

The first article introduces micro computed tomography (µCT). In a review paper, Handschuh and Glösmann offer excellent insights into the art of sample fixation, sample mounting, tissue contrasting and image analysis. They also put the theoretical consideration in praxis and provide impressive examples of qualitative and quantitative analysis of mouse embryo morphology.

In the following article, Scully and Larina switch to optical coherence tomography (OCT). They demonstrate the capability of this non-invasive technique for classical structural embryo phenotyping and pay special attention to the potential of OCT to study dynamic events by live phenotyping of cultured early mouse embryos. Technical limitations as well as future perspectives and the potential to become integrated in multimodal imaging approaches are comprehensively discussed.

Next, Anderson and Bamforth show results of using two cutting edge imaging techniques, “High resolution episcopic microscopy” (HREM) and µCT for gaining insights into basic morphogenetic events. Their article “Morphogenesis of the Mammalian Aortic Arch Arteries” visualizes and examines remodeling of the embryonic pharyngeal arch arteries in mice and men. It adds new details to the knowledge about the formation of the external carotid and subclavian artery, and to the role *Tbx1* plays in the genesis of cardiovascular defects.

A contribution by Garcia-Canadilla et al. follows, where they apply structure tensor analysis on HREM-derived volume-data for characterising and for quantifying the myocardial architecture of mouse embryos at embryonic days (E)14.5 to E18.5. The quantitative results demonstrate gradual increase of the complexity of myocardial organization with developmental progress and a regional heterogeneity in myocardial architecture.

HREM data are also used in the article of Reissig et al. The authors carefully analysed cranial nerve topology in volume data of 152 C57BL/6 wild type mouse embryos produced in the “Deciphering the mechanisms of developmental disorders” (DMDD) program. The information was then used as a reference for diagnosing cranial nerve abnormalities in 4 knock-out lines produced in the DMDD project and for defining the range of norm variants in wild types.

An article of Petrelli et al. follows, in which the authors rely on an interesting mixture of imaging modalities for characterising a new mouse model created to study the mechanisms underlying Foetal Alcohol Syndrome Disorder (FASD). µCT, scanning electron microscopy, whole-mount *in situ* hybridization, and immunohistochemistry were applied to create data that allowed the detection of a number of craniofacial malformations recapitulating defects characteristic of prenatal alcohol exposure.

Finally, the review by Copp et al. demonstrates the merits of performing phenotyping of cultured embryos with the aid of stereomicroscopy. Besides providing imaging tips, they direct the reader to an innovative and largely unknown whole mouse embryo culture approach, which enables experimentation with mammalian embryos.

We like to express our gratefulness to all the imaging specialists who contributed to this Research Topic and hope that the embryo phenotyping community will profit from this Research Topic of highly interesting articles.

